# Hospitalization of the aged due to stroke: An ecological perspective

**DOI:** 10.1371/journal.pone.0220833

**Published:** 2019-08-07

**Authors:** Marcelo de Jesus Carlos, Ana Carolina Lima Cavaletti, Célia Pereira Caldas

**Affiliations:** 1 Postgraduate Program in Medical Sciences, Medical Science College, Rio de Janeiro State University, Rio de Janeiro, Rio de Janeiro State, Brazil; 2 Department of Aging, Nursing School, Rio de Janeiro State University, Rio de Janeiro, Rio de Janeiro State, Brazil; University of Malaya, MALAYSIA

## Abstract

Contextual variables have been associated with the incidence of stroke, but their association with hospitalization of older persons remains unclear. This study evaluated the association between social context variables and hospitalization of 60 years old and older patients due to stroke in Rio de Janeiro, Brazil. An ecological cross-sectional study was conducted, with secondary data from the Brazilian Hospital Information System from 2006 to 2014. Hospitalization rates were calculated and categorized by tertiles. For subsequent analyzes, the polar extremes method was used to select the groups with extremes values. After that, Student t or Mann-Whitney tests were used to compare the contextual variables and the hospitalization rates clusters. Then, a Binary Logistic Regression analysis was used to assess the association between hospitalization rates clusters and the contextual variables. The total number of hospitalizations was 82 796; the hospitalization rate varied in extremes groups from the lowest (3.49) to the highest (11.95) (p<0.001). The highest rates group was positively associated with the proportion of elderly (p<0.001), the illiteracy rate of the aged (p = 0.01), primary care coverage (p<0.001) and ambulatory care for hypertension and diabetes, while the income ratio showed negative association with the highest rates of hospitalization (p = 0.01). In the multivariate analysis, only the proportion of elderly (OR = 1.55; 95%CI 1.07–2.25), primary care coverage (OR = 1.05; 95%CI 1.01–1.11) and income ratio (OR = 0.82; 95%CI 0.67–0.99) maintained the association. In conclusion, contextual variables in the three dimensions studied were associated with the rate of hospitalization of aged due to stroke in the municipalities in Rio de Janeiro State. Transitional care and other improvements in both the health care and social services are demanded.

## Introduction

Social disadvantage affects both intellectual and social development. Those who are privileged have better performance in measures of development due to the circumstances of social inequity [1]. Nowadays, there is a worldwide interest in identifying and solving the gaps in health inequities; especially those related to the impact of contextual factors on health and disease processes [2,3]. At the ecological level, the environment, culture, demographic and socioeconomic characteristics are factors that can impact the health and disease profile of the population, as well as the access to preventive and health care [3,4].

In absolute terms, the incidence of stroke has decreased in recent decades. In spite of it, due to population growth and aging, the incidence rate continues to progress rapidly in developing countries. Furthermore, stroke is a global health problem; it is the second most disabling disease in developing countries, and the third in developed countries [5].

In Brazil, stroke remains a neglected disease and the social and regional differences within the country are important factors in making this happen. Access to knowledge and means for prevention, treatment and rehabilitation is different, according to the social class and the region of the country [6,7]. Nevertheless, considering that stroke is a primary care sensitive disease, the incidence and hospitalizations may be reduced through preventive policies [8].

Due to the high incidence in the elderly [9], stroke is one of diseases that most influence the loss of quality of life in this population. Hospitalization is a major aggravating factor [10]. In addition, aged persons are the majority of users of public health system [11]. Therefore, understanding the determinants of hospitalizations caused by stroke in aged persons in the ecological context is important to identify the needs of preventive policies for Primary Care [12].

Some Brazilian studies have had also shown interesting results both in socioeconomic dimension and in other dimensions of health determinants [13,14], however ecological studies on hospitalization of the aged in Rio de Janeiro State are needed [15]. The aim of the present study was to investigate the association of contextual variables and hospitalizations due to stroke in Rio de Janeiro State, Brazil.

## Materials and methods

### Data

The Rio de Janeiro State is a unit of the Brazilian Federative Republic situated in the Southeastern region with a population of 15,989,929 inhabitants. Regarding geopolitics, the State is divided into 92 municipalities.

A cross-sectional ecological study was performed with data from the Hospital Information System of Brazilian National Public Health System. The researched period was January 1, 2006 to December 31, 2014. This period covers four years before and after the 2010 Brazilian Demographic Census. Municipal level was the operational basis, and hospitalizations data were extracted according to the codes of International Classification of Diseases, tenth revision (ICD-10).

The total number of hospitalizations registered from 2006 to 2014, according to the municipalities of residence was divided by the total number of residents 60 years old and older and multiplied by 1000. The number of the elderly residents enrolled in 2010 Census was used for estimating the total population in the researched period. Subsequently, hospitalization rates were divided by nine, which is the number of years included in the cross section. Therefore, the rate of hospitalization of aged due to stroke refers to the unit 1000 persons/year.

The contextual variables refer to the demographic, socioeconomics and health information of municipalities. Regarding the demography, the proportion of the oldest old (percentage of the elderly people aged 85 and older) and the aged gender ratio were chosen (the number of the aged man older than 60 for each group of 100 women for the same range). About the socioeconomics conditions, the income ratio (number of the times the income of the richest 20% is higher than of the poorest 20%), and the illiteracy rate of the aged (percentage of individuals aged 60 and older who cannot read and write a simple ticket in Portuguese) were evaluated. The health variables were the primary care coverage (percentage of individuals enrolled in public primary care) and the rate of hypertensive and diabetic patients follow-up (percentage of individuals diagnosed with systemic arterial hypertension and diabetes mellitus in primary care). The demographic and socioeconomic variables were collected from the database of Brazilian Institute of Geography and Statistics, and refer to 2010 Census and from Information System of Brazilian national public health system.

### Inclusion and exclusion criteria

Hospitalizations of residents 60 years old and older, living in municipalities of Rio de Janeiro State, coded as stroke (ICD-10; I.60-I.66) and transient ischemic attacks (G.45) were included. As the diagnoses were established at the time of admission, we included Transient Ischemic Attacks (TIA) due to the difficulty in differential diagnosis [16]. In the analysis, municipalities with outlier data were excluded.

### Statistical analysis

We performed the univariate analysis of the dependent (the hospitalization rates due to stroke for 1000 person/year) and independents variables (contextual variables), through its standard values (Z scores), in order to find univariate outliers. After excluding the univariate outliers that presented a Z score above 3 standard deviations, all the quantitative variables were placed in a linear regression model to identify the multivariate outliers, through the Mahalanobis distance.

Then, the municipalities were categorized into tertiles of hospitalization rate. The tertiles I (low hospitalization rates) and III (high hospitalization rates) were chosen for association analysis using the polar extremes method, excluding the 31 municipalities that belonged to the tertile II. After the categorization of the variable, a new exploratory analysis was carried out for all the contextual variables with the objective of evaluating the distribution of data within each cluster (tertiles I and III) through the Shapiro-Wilk test. The variables that did not present normal distribution were represented by their medians and interquartile range and those that presented such distribution, by their means and 95%.confidence interval (95%CI).

Subsequently, the collinearity and multicollinearity diagnoses were performed among the independent variables. Collinear variables were considered those with a correlation coefficient greater than 0.7 in the bivariate analysis using the Pearson or Spearman tests and as multicollinear variables with variance inflation factor greater than 10.

The comparison of each contextual variable between the two groups of hospitalization rate was performed through the Student t or the Mann-Whitney tests, according to the distribution of the data. Contextual variables that did not present a statistically significant difference (p <0.05) were not included in the logistic regression model.

Finally, the logistic regression model was performed through of the insertion method of the contextual variables to the base model. The value considered statistical significant was 2 LL of the likelihood between the base model and the proposed model, explaining the value of the pseudo coefficient of determination and the percentage of municipalities correctly classified in each group. In addition, all variables of the model participated as co-variables in the analysis of each contextual variable. The association’s magnitude was verified by the Odds Ratio (OR) in a 95% confidence interval, considering the group of low hospitalization rates as a reference group (tertile I).

## Results

From 2006 to 2014, 124,712 hospitalizations for stroke occurred in the state of Rio de Janeiro, of which 82,796 were hospitalizations of aged persons. Almost half (50.49%) were women, with 37.62% in the 70–79 age group. A percentage of 64.66% did not receive an accurate diagnosis of stroke or TIA subtype at admission (Table 1).

**Table 1:** Hospitalizations of the aged due to stroke, by sex, age group and subtype of stroke.

Descriptive variables	Hospitalizations	
	Number	Percent
**Sex**		
Men	40 995	49.51
Women	41 801	50.49
**Age groups**		
60–69	30 366	36.68
70–79	31 149	37.62
80 or older	21 281	25.70
**Stroke subtype**		
Ischemic	7 174	8.66
Hemorrhagic	9 542	11.53
TIA	12 543	15.15
Non-specified	53 537	64.66
Total	82 796	100

Two municipalities with Z score above 3 were identified as outliers and excluded from the sample. The municipalities of Itaocara (Z = 3.64 in hospitalization rate) and Rio de Janeiro City (Z = 3.41 in income ratio) were excluded from analysis. No multivariate outliers were found.

Comparing the hospitalization rates in each cluster, municipalities with higher rates (tertile III) had 3.42 more times hospitalizations due to stroke per 1000 person-year. Five contextual variables presented statistically significant differences in relation to hospitalization rates clusters. Table 2 shows the analyses of the contextual variables between the two clusters.

**Table 2:** Comparison of the independent variables among the groups of municipalities hospitalization rates.

Variables	Tercile I n* (29)	Tercile III n* (30)	p
Hospitalization rate (95% CI) †	3.49 (2.95 to 4.02)	11.95 (11.15 to 12.74)	< 0.001
Aged sex ratio (95% CI)	0.83 (0.79 to 0.87)	0.86 (0.82 to 0.89)	0.223
Proportion of oldest old IR (Q_25_ to Q_75_) ‡	11.90 (11.50 to 14.15)	15.40 (13.90 to 16.23)	< 0.001
Illiteracy rate among the aged IR (Q_25_ to Q_75_)	18.20 (12.05 to 22.75)	25.25 (17.58 to 28.40)	0.014
Income ratio IR (Q_25_ to Q_75_)	17.46 (15.37 to 20.61)	15.23 (13.36 to 17.13)	0.012
Primary care coverage IR (Q_25_ to Q_75_)	70.85 (40.77 to 98.81)	99.16 (79.60 to 100)	< 0.001
Hypertensive and Diabetic outpatient follow-up rate IR (Q_25_ to Q_75_)	34.71 (20.21 to 67.20)	104.82 (70.00 to 146.70)	0.001

*n number of municipalities in each group.

^†^ 95% CI 95% confidence interval of the mean.

^‡^ IR (Q_25_ –Q_75_) Interquartile range.

The logistic regression model with the five contextual variables presented a predictive accuracy of 78% of correctly classified municipalities within the model. In addition, there was a statistically significant variation in the likelihood between the base model and the general model (p <0.001), with a pseudo coefficient of determination of 0.514. In the model quality diagnosis through the analysis of residues, we did not find misclassified cases.

After carrying out the logistic regression model, three contextual variables remained statistically significant in the association with higher hospitalization rates cluster. The income ratio showed negative association (OR 0.82 95% CI 0.67–0.99), while the proportion of oldest old (OR 1.55, 95% CI 1.07–2.25) and the primary care coverage (OR 1.05, 95% CI 1.01–1.11), had positive association (Table 3).

**Table 3:** Association between the contextual variables with higher hospitalizations rate of aged due to stroke cluster. The Reference group is tercile I (low admission rate).

Contextuable variables	OR*	95%CI†	IRt‡
Proportion of elderly	1.55	1.07 to 2.25	55%
Illiteracy rate among the aged	0.96	0.85 to 1.08	- 4%
Income ratio	0.82	0.67 to 0.99	- 18%
Primary care coverage	1.05	1.01 to 1.11	5%
Hypertensive and Diabetic outpatient follow-up rate	1.00	0.98 to 1.02	0%

*OR Odds ratio.

^†^95%CI 95% Confidence interval.

^‡^IRt Inequality ratio.

Proportion of oldest old was the variable with highest magnitude of association. The one-point increase in this variable increases the distance between the groups rates by 55%, while the income ratio decreases this distance by 19%.

## Discussion

Brazil has achieved, in recent years, effective results regarding the reduction of poverty and of income disparities and the expansion of health coverage. However, inequalities in access to services and health outcomes remain a major challenge. [17].

This study has shown significant variation in hospitalization rates due to stroke in 60 years old and older persons in Rio de Janeiro State. We consider these rates as health outcomes that are expressed differently according to the context in which they occur due to the strong causal link between income disparities and health issues [18].

In Brazil, income disparities have already been positively associated with the increase in mortality rates due to stroke [17]. This variable was shown to be a relevant determinant of health for middle-income and low-income countries [19].

This study showed a gap of 8.46 hospitalizations per 1000 person-years among municipalities with lower and higher hospitalization rates, suggesting the impact of social context in this health outcome. In regions with lower economic development, specific factors such as resource availability, cultural beliefs and population expectations about health services can influence the number of diagnosed cardiovascular diseases [20].

Most hospitalizations occurred in persons under 80 years old. Furthermore, the proportion of 60 years old and older persons was the variable with strongest association with the rates of hospitalization due to stroke. This result expresses the challenge policymakers and public managers need to face in order to offer a friendly environment and structured health services to meet the specificities of this population [21].

The association of the proportion of 60 years old and older persons with higher hospitalization rates explained 55% of the logistic regression model in the present study. The municipalities in first tertile have lower proportion of 60 years old and older persons, despite its greater income inequality. The populations of these municipalities are more educated, consistent with the higher average per capita income. They have greater access to information, to consumer goods, greater offer of health services and greater technological apparatus, both in professional training as inputs. In addition, these municipalities have greater range of private services that are accessible to those with higher incomes [22].

In Rio de Janeiro State, municipalities with highest income disparities are the most urbanized. They have less population living in rural areas and greater coverage of private health systems [22]. This may explain the negative association between hospital admissions in public health system and municipalities with highest income disparities.

Population aging is a challenge for health systems worldwide, especially in developing countries. Studies show the prevalence of older persons living with multi-morbidities [23], and the precocity of this health condition in populations living in social deprivation areas [24]. This is a result of population aging without socioeconomic and living conditions improvements.

The association between proportion of elderly and municipalities with high rates of admissions due to stroke was found, as expected. Elderly patients with a high number of multiple morbidities are in the oldest group and are vulnerable to diseases of all kinds. In addition, elderly who do not have health insurance, who depend exclusively on public health system and who live in areas covered by the family health strategy, are the ones that most present morbidities related to cardiovascular disease risk factors. This fact may confirm a social concern, since this population tends to be less educated and more susceptible to socioeconomic vulnerabilities [25].

Municipalities with high rates of admission presented higher rates of illiteracy among the aged. However, within the model with the other covariables the association lost the strength.

Individual and ecological studies have shown a negative association between the incidence and mortality due to stroke with educational levels of the population, both in Brazil and in more economically developed countries [26–28]. This social disadvantage affects both, intellectual and social development of population in general. There is a gradient: less disadvantaged societies have better development measures [1].

In Brazil, education plays a positive role in relation to behavioral risk factors of cardiovascular diseases. Populations with higher levels of education are more protected against tobacco use, tend to eat healthier and their individuals have more time to practice physical activity, although they prefer a more sedentary lifestyle [29].

Despite lower income inequality, municipalities that presented a lower rate of stroke have lower per capita income, less access to services and consumer goods, which exposes the population to a context of limitations [22].

The variation of hospitalization rates among the most prevalent cardiovascular diseases can be partially attributed to contextual risk factors and can be explained by the different profiles of the analyzed spaces. Reducing community risk factors in the long term can result in a significant reduction in hospitalizations due to these diseases.

Different parameters comprise spacialization and this includes both characteristics of the inhabitants and the built environment. Improving spatial characteristics can reduce the prevalence of cardiovascular risk factors. The regional variation in admission rates and risk factors show the complex connection between space and health, and to understand this interaction is paramount for the development of preventive strategies [30].

It should be noted that many improvements observed in national health indicators stem from the many investments in social policies during the years 2003 to 2016, which, in synergy with various health policies and actions, contribute to the improvement of the population situation [31].

In Brazil, there is a beneficial effect of primary care coverage on stroke-related mortality and hospitalization rates for other cardiovascular diseases [32]. In addition, there is evidence of a better prognosis of the disease in areas covered with basic care [8,33].

Different degrees of functioning of public health system and implementation of local primary health care is a reflect of what was found in this study: the greater chance of municipalities with higher coverage of primary health care and higher rates of outpatient follow-up of hypertension and diabetes mellitus belong to the group of high hospitalization rates in the period studied.

Primary care coverage is associated with the management of conditions sensitive to primary care and consequent reduction of hospitalizations. However, the expansion of access to services began in the 1990s through the Family Health Program, which later became a health strategy (Family Health Strategy). As one of the ways of reducing iniquities, the Family Health Strategy covers mainly low-income, lower-educated level, and disadvantaged municipalities [31].

In addition, the administration of these services is a responsibility of the municipalities, which do not always have scientific and managerial technical knowledge, adequate structure and health professionals in sufficient quantity and capacity to operate in their territory [34].

The percentage of hospitalizations without accurate diagnosis is an example. It can be explained by the unavailability of a CT scanner in hospitals [35,36], by insufficiency of neurologists in emergencies of public health system impacting on the reduction of quality in the diagnosis of the stroke subtype [37] and by the quality of records regarding the subcategories of ICD-10 [36,38].

On the other hand, in Brazilian public health system, the indifference when coding the disease can also be explained by, despite the efforts to train professionals to properly fill the records, the refunding process is prioritized; not the accuracy of diagnosis [38].

Thus, besides the challenge to meet the demand with an accumulated burden of illness, primary care level has the role of coordinating a health system regulated by a health policy still under construction. [39–41].

### Limits

There are some limits to this study. As regards to internal validity, the Hospitalizations information System from Public Health system (SIH-SUS) is targeted to be the payment system for hospital services and not to identify readmissions or transfers from other hospitals due to the same cause of hospitalization. Besides that, the data is based on secondary data and may have sub-registration. The coding of records depends on the competence of doctors at the moment of hospitalization, being influenced by their clinical knowledge or factors related to service management [31].

The Brazilian census is held every 10 years. The collected data presents the demographic and socioeconomic situation of population in the year of collection. The variation in the characteristics of the population, occurring in the gap between censuses, expresses the impacts of the actions and policies in the past. They are also a reference for guiding policies in the coming years. Thus, when we use contextual data from the year 2010 (middle of the period between 2006 and 2014), we are considering as an estimation of the context in the period of this study.

We understand that the behavior of searching for hospital services is multifactorial and individual. We also understand that access to and provision of such services exerts an influence on individual behavior [42,43]. However, the variable rate of hospitalization for stroke in this study is a proxy for health outcome (in municipalities population).

This is an ecological study, which is differentiated by the exposure measurement method (collective data only) and the grouping method. Ecological studies assess how the social and environmental context can affect the health of population groups and inferences cannot be considered at the individual level. Extrapolating the inferences to the individual level would be a misconception called ecological fallacy.

## Conclusion

We found a high proportion of hospitalization due to stroke in Rio de Janeiro State. However, the rates vary significantly among groups of municipalities with different characteristics. The municipalities with the lowest income inequality are also the poorest and have a higher percentage of illiterate elderly people. These municipalities are at the tercil with higher hospitalization rates.

We found lower admission rates in municipalities with higher income inequality, where the population is more educated and the average income per capita is higher. In these places, there is greater availability of services and consumer goods, besides of the private health services for those who can pay.

The strong positive association between inpatient rates and the proportion of older people expresses the challenge for governments. Further efforts and investment are needed in regions where the rise in life expectancy has not been accompanied by social improvements. And, the positive association between primary care coverage and hospitalizations due to stroke is a little against common sense, while this also indicates the health service alone cannot solve the health demands of a population with accumulated illness. Transitional care and other improvements in both the health care and social services are demanded.
